# Successful cutting balloon angioplasty in a child with resistant renal artery stenosis

**DOI:** 10.1186/s13104-015-1673-z

**Published:** 2015-11-12

**Authors:** Jae Sung Son

**Affiliations:** Department of Pediatrics, Konkuk University Medical Center, Konkuk University School of Medicine, 120-1 Neungdong-ro, Gwangjin-gu, Seoul, 05030 Korea

**Keywords:** Renal artery stenosis, Fibromuscular dysplasia, Cutting balloon angioplasty, Child

## Abstract

**Background:**

Although renovascular hypertension is a rare disease, it is associated with 5–10 % of cases of childhood hypertension. It is a potentially treatable cause of hypertension, and is often caused by renal artery stenosis (RAS). The most common cause of RAS in children is fibromuscular dysplasia (FMD). The options for treating RAS depend on the location, severity and abnormality underlying the condition.

**Case presentation:**

A previously healthy 7-year-old Korean boy presented to our clinic with hypertension and headache. Renal ultrasonography and multi-detector computed tomography (MDCT) showed severe focal stenosis at the middle portion of the left renal artery (LRA) and multiple collateral vessels. Percutaneous balloon angioplasty was performed as an initial treatment, but yielded unsatisfactory results. The presence of intimal-type FMD was suspected based on his clinical features, angiographic appearance, and resistance to percutaneous transluminal renal angioplasty. Thereafter, his blood pressure was normalized using antihypertensive medication. Follow-up multi-detector computed tomography at 11 years of age showed persistent severe stenosis of the LRA. After unsuccessful attempts to perform balloon angioplasty, 3.5-mm cutting balloon angioplasty (CBA) was performed and yielded satisfactory results. He was discharged without any medication. At 1 year and 6 months after the intervention, he has been normotensive and had not required any antihypertensive medication.

**Conclusion:**

The author describes a case of resistant RAS that was detected on MDCT and successfully treated using percutaneous (CBA). Although cutting balloon angioplasty is useful in many clinical conditions, including the current case, clinicians should carefully consider the associated risk of arterial disruption and pseudoaneurysm formation.

## Background

Although renovascular hypertension (RVH) is a rare disease, it is an important cause of childhood hypertension, and serves as a causative factor in 5–10 % of all cases of childhood hypertension [1]. It is a potentially treatable cause of hypertension, and is often caused by renal artery stenosis (RAS); however, it is difficult to diagnose and treat in children. Conventional contrast angiography (CCA) remains the gold standard for the evaluation of the renal artery, but multi-detector computed tomography (MDCT) also enables to provide anatomical evaluation at a high resolution with a short scan time [2, 3]. The most common cause of RAS in children is fibromuscular dysplasia (FMD); this can be treated by percutaneous transluminal renal angioplasty (PTRA), with a high technical and clinical success rate [4]. As PTRA is less invasive and more cost-effective alternative to open surgery and can be performed with a high degree of technical success with minimal complications, PTRA with a conventional balloon catheter has become the primary choice for revascularization, particularly in cases caused by FMD. However, some lesions are resistant to PTRA with conventional balloons and typically require open surgery. Following technical advancements, cutting balloon angioplasty (CBA) was recently introduced, and may serve as a useful treatment option for RAS resistant to PTRA with conventional balloons in children. A conventional balloon tears the stenotic arterial wall, whereas the cutting balloon—which has 3–4 longitudinal blades mounted on the outer surface—creates an incision in the vessel wall with these blades, reduces elastic recoil, and can improve the technical result and possibly long-term patency [2, 5]. In the present report, the author describes a case of resistant RAS that was suspected to be caused by FMD, was confirmed by MDCT, and was successfully treated with CBA.

## Case presentation

**Fig. 1:**
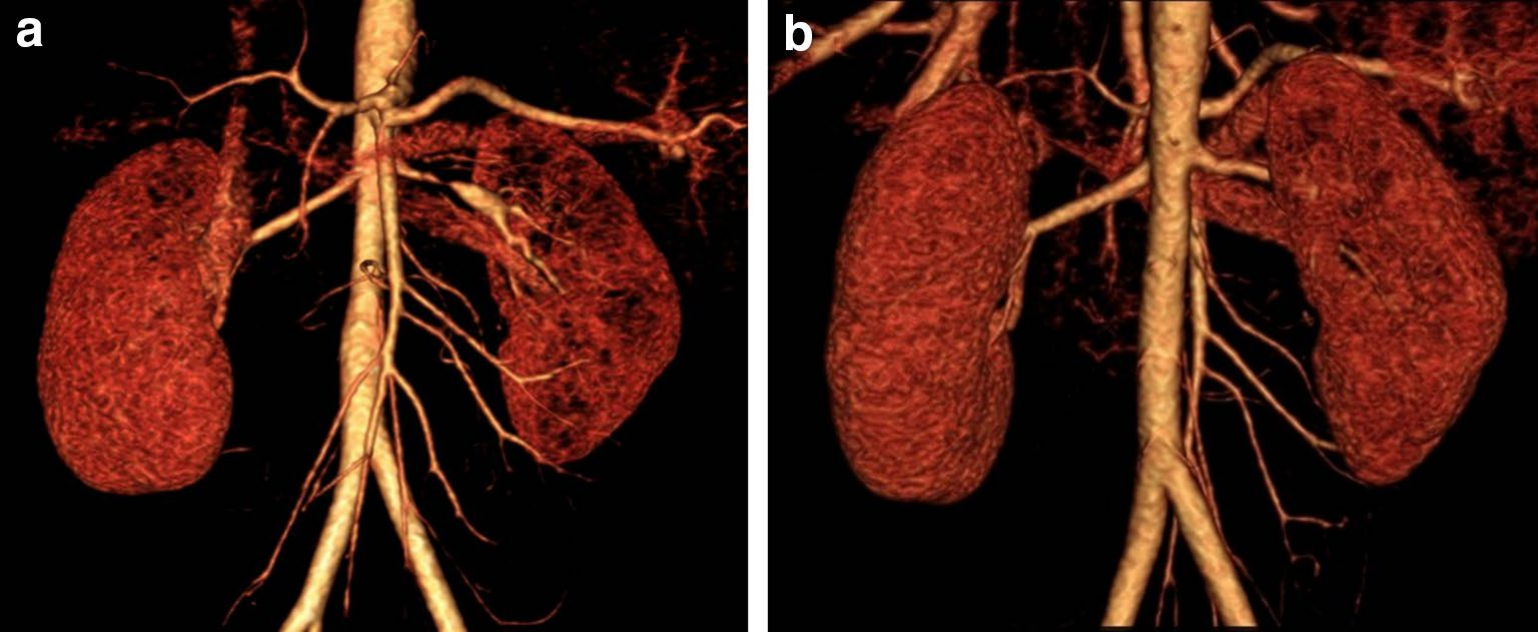
Three-dimensional multi-detector computed tomographic angiogram. **a** Severe focal stenosis is observed in the proximal area of the left renal artery on pre-mapping with 64-channel multi-detector computed tomographic angiography, before the interventional procedure. **b** Dilated and patent renal artery stenosis is observed at 7 months after cutting balloon angioplasty

**Fig. 2:**
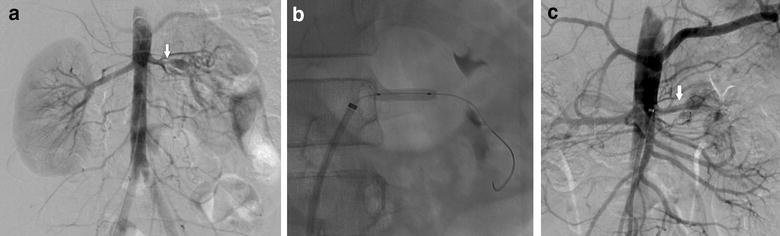
Abdominal angiogram before and after cutting balloon angioplasty. **a** Abdominal aortogram shows a severe focal stenosis with post-stenotic dilatation in the proximal area of the left renal artery (*arrow*). **b** A 3.5-mm cutting angioplasty balloon is inflated, an incision is made successfully, and the stenotic region is dilated. **c** Abdominal aortogram shows a successfully dilated, patent left renal artery after cutting balloon angioplasty (*arrow*)

A previously healthy 7-year-old Korean boy presented to our clinic with hypertension and intermittent headaches. His blood pressure (BP) was 137/93 mmHg and 24 h ambulatory mean BP was 181/117 mmHg (daytime, 197/125 mmHg; nighttime, 160/91 mmHg). He was admitted for further evaluation of the hypertension. He had no episodes of persistent fever of unknown origin, and no symptoms such as stomatitis, arthritis, and skin rash that were indicative of autoimmune disease. There was no family history for autoimmune, cardiovascular, or renal disease. Except for the high BP values, the physical examination yielded unremarkable results. He also didn’t exhibit any facial anomalies or skin lesions. Laboratory examination indicated that blood cell count, serum creatinine levels, serum electrolyte levels, thyroid function, and urinalysis results were normal. In addition, serum C-reactive protein (CRP) levels and erythrocyte sedimentation rate (ESR) were normal. Vasculitis screening, including tests for antinuclear antibodies, antibodies to double-stranded DNA, and anti-neutrophil cytoplasmic antibodies, indicated normal results. Fundoscopic examination showed minimally increased vascular tortuosity. Echocardiography showed no specific abnormalities. Renal ultrasonography demonstrated abnormal Doppler waveforms in the left kidney, characterized by a tardus–parvus pattern [6]. A 64-channel MDCT showed severe focal stenosis at the middle portion of the left renal artery (LRA) and multiple collateral vessels. PTRA was performed with multiple inflations; during the final attempt, the balloon was inflated at a pressure beyond the burst pressure. However, unsatisfactory results were obtained. The author suspected a diagnosis of intimal-type FMD based on his clinical features, angiographic appearance, and resistance to PTRA. As his BP could be well controlled with antihypertensive medication after the initial intervention, the author decided to attempt PTRA once more or another surgery after he reached puberty. A repeat MDCT (Fig. 1a) at 11 years of age showed persistent severe stenosis at the middle portion of the LRA with post-stenotic dilatation. Abdominal angiography (Fig. 2a) showed similar results, and hence, PTRA was attempted 3 times by using high pressure balloons with different sizes. However, as the dilatation was not successful and the levels of inflammatory markers (ESR and CRP) were within normal range, we decided to attempt percutaneous revascularization via CBA. After CBA was performed with a 3.5-mm cutting balloon (Fig. 2b), repeat abdominal angiography (Fig. 2c) showed no clinically significant residual stenosis. He was discharged without any antihypertensive medication. At 7 months after CBA, on follow-up MDCT, no signs of re-stenosis of the LRA were noted (Fig. 1b). Subsequently, at 1 year and 6 months after CBA, he appears normotensive and has not required any antihypertensive medication.

## Discussion

RVH remains a diagnostic and therapeutic challenge in the pediatric population [7]. Various diseases, including FMD, developmental RAS, neurofibromatosis type 1 (NF-1), Moyamoya disease, vasculitis such as Takayasu’s disease, Alagille syndrome, Marfan syndrome, and Williams syndrome, are associated with pediatric RVH [1, 8]. FMD is an idiopathic angiopathy characterized by non-atherosclerotic and non-inflammatory fibroplastic disease that most commonly affects the renal and carotid arteries, and is the most common cause of pediatric RAS [9, 10]. In general, it is four times more prevalent in females than in males in adults, although the sex distribution in children is equal [8]. FMD may be histologically classified based on the arterial wall layer involved—i.e., medial (70 % of cases), perimedial (15–25 %), and intimal fibrodysplasias (1–2 %) [11]. The intimal type of FMD usually presents with a highly stenotic, usually proximal, localized lesion with post-stenotic dilatation. Angiographic features of the intimal type of FMS are more commonly noted on angiography in children than in adults. Both the medial and perimedial types of FMD manifest as alternating stenosis and aneurysms, such that they appear as a “string of beads” on angiography. However, this classic “string of beads” pattern is not frequently observed on angiography in children [1, 2, 8]. As FMD is often a suspected diagnosis or diagnosed based on exclusion, the distinct clinical features between children and adults should be clearly determined. In particular, the medial type of FMD is less commonly encountered in children. In contrast, the intimal type of FMD is uncommon in adults, but is the most common type in children [9]. Among the three histologic types of FMD, dilation is reported to be more difficult in the intimal type than in the medial or perimedial types. FMD is diagnosed based on the angiographic appearance and following the exclusion of other conditions. In the present case, although the patient could not be diagnosed based on the results of histological examinations, we suspected a diagnosis of intimal-type FMD, based on the patients’ history, laboratory results, angiographic appearance (focal stenosis in the middle of the renal artery with poststenotic dilatation, and no characteristic beaded pattern), and resistance to PTRA. Although the technical success rate of pediatric PTRA is high, the long-term success rate of pediatric PTRA is lower than that in adult; this difference may be due to the presence of a smaller vessel diameter and a more pronounced response to growth factors in immature vasculature [10]. Several imaging modalities such as CCA, Doppler ultrasonography, renal scintigraphy, computed tomography angiography (CTA), and magnetic resonance angiography (MRA) have been used to evaluate the renal artery. CCA is the gold standard for the diagnosis of RAS and is especially useful in the evaluation of branch vessel disease. However, CCA requires arterial catheterization and the use of general anesthesia in young children, and is associated with a small risk of complications. CTA is a non-invasive technique that does not require arterial contrast injection, and reportedly has equivalent accuracy as that of MRA in adult patients [3]. MDCT can provide more detailed information for the three-dimensional visualization of RAS, with a shorter scan time, less respiratory motion artifacts, and high resolution, in a non-invasive manner [3]; moreover, the associated cost and ionizing radiation exposure is lower than those associated with CCA [3, 12]. This could facilitate the planning of the interventional procedure, and may shorten the catheterization time and reduce the contrast injection dose required. Due to these advantages, CTA is currently used for pre-mapping, before the interventional procedure, in some centers [8]. The current day-to-day medical practice guidelines recommend that FMD should be diagnosed based on the CTA or MRA findings. FMD may also be diagnosed using CCA (the gold standard), in cases where FMD is strongly suspected but the non-invasive test results are inconsistent. During follow-up periods, CTA may also be useful to evaluate the effect of the therapies [13]. At present, PTRA is considered the treatment of choice for RAS as a result of FMD, and has a high technical and clinical success rate [4, 7]. The treatment of RAS with PTRA may be difficult because of arterial wall thinning, friability, and the firm elastic recoil of the intramural lesion in some conditions such as NF-1, William syndrome, or mid-aortic syndrome. In few cases, PTRA may not be able to dilate the RAS, and hence, the success rates may vary from 48 to 90 % [7, 9, 14]. Even if it is not curative, PTRA may play a role in temporarily managing hypertension in younger children prior to surgical repair scheduled during puberty. In the present case, as the hypertension was well controlled with anti-hypertensive medication after initial PTRA, the decision to perform either re-intervention or open surgery was delayed until puberty, when the procedure can be performed with better long-term results, even though the result of initial PTRA was unsatisfactory. CBA is reportedly effective for treating stenotic lesions related to FMD that are resistant to conventional balloon angioplasty, particularly in cases of stenosis related to intimal FMD [2]. At present, cutting balloons are available in a wide variety of diameters and lengths, with a range suitable for the treatment of pediatric lesions, and have improved the technical success rate of PTRA. The complications associated with CBA in the treatment of RAS are similar to those for PTRA, including dissection, rupture, and thrombosis of the renal artery [15]. Although CBA extends the therapeutic potential for resistant RAS to small children, it is not a panacea and clinicians should not expect successful results in all children.

## Conclusion

FMD is the most common cause of RAS in children. PTRA is a well-established therapy for RAS in adults and children, but conventional balloon angioplasty is ineffective in some patients. CBA is a safe and effective alternative to surgical therapy for resistant RAS. Although CBA is useful for the treatment of many clinical conditions, clinicians should not consider it an absolute treatment option and should not expect success in all children, as the efficacy and long-term effects of this procedure have not yet been established.

## Consent

Written informed consent was obtained from the patient’s parents for publication of this case report and any accompanying images.

## Abbreviations

RVH: renovascular hypertension
RAS: renal artery stenosis
CCA: conventional contrast angiography
MDCT: multi-detector computed tomography
FMD: fibromuscular dysplasia
PTRA: percutaneous transluminal renal angioplasty
CBA: cutting balloon angioplasty
BP: blood pressure
CRP: C-reactive protein
ESR: erythrocyte sedimentation rate
LRA: left renal artery
NF-1: neurofibromatosis type 1
CTA: computed tomography angiography
MRA: magnetic resonance angiography
